# *Vitis labrusca* Extract (HP01) Improves Blood Circulation and Lipid Metabolism in Hyperlipidemic Rats

**DOI:** 10.1155/2020/6180310

**Published:** 2020-12-24

**Authors:** Bo Yoon Chang, Dae Sung Kim, Sung Yeon Kim

**Affiliations:** ^1^Institute of Pharmaceutical Research and Development, College of Pharmacy, Wonkwang University, Iksan, Jeonbuk 54538, Republic of Korea; ^2^HanpoongPharm.Co., Ltd., 333-24 1st Palbok-dong, Deokjin-gu, Jeonju-si, Jeonbuk 561-841, Republic of Korea

## Abstract

Excessive intake of high-lipid foods and lifestyle changes can easily cause hyperlipidemia. Hyperlipidemia is clinically considered a major risk factor for cardiovascular disease, which is the second leading cause of death worldwide. In this study, the effects of a *Vitis labrusca* extract (HP01) on coagulation, platelet aggregation, and lipid metabolism were investigated in hyperlipidemic rats. A rat model of high-fat diet- (HFD-) induced hyperlipidemia was used. Hemostatic parameters and lipid levels were investigated after HP01 treatment of hyperlipidemic rats. Different doses of HP01 (200 mg/kg/day and 400 mg/kg/day, p.o.) were administered for 3 weeks, and prothrombin time (PT), activated partial thromboplastin time (aPTT), and platelet aggregation and bleed time (BT) were determined. The levels of thromboxane B(2) (TXB(2)) and serotonin were measured using enzyme-linked immunosorbent assay kits. Simultaneously, hepatic function and blood fat indexes, including aspartate aminotransferase (AST), alanine aminotransferase (ALT), triglyceride (TG), malondialdehyde (MDA), and glutathione (GSH), superoxide dismutase (SOD), catalase (CAT), and glutathione peroxidase (GPx) were also measured. In comparison with the data obtained for rats in the untreated HFD group, HP01 (200 mg/kg) treatment prolonged PT but did not affect aPTT. HP01 treatment did not alter plasma TXB(2), PGI2, or serotonin levels. However, HP01 showed some effects in improving liver function by reducing the levels of hepatic lipids. ALT, MDA, and hepatic TG levels significantly decreased, whereas GSH, GPx, CAT, and SOD levels significantly increased. These results confirm the HP01 extract will improve thromboplastic and the liver metabolic disorders in hyperlipidemia by oxidative stress response.

## 1. Introduction

The incidence of heart and vascular diseases is increasing everyday because of the recent improvement in living standards, westernized dietary habits, and changes in living standards. Cardiovascular disease (CVD), which describes all conditions affecting the heart and blood vessels, is currently the leading cause of death worldwide. There are several risk factors associated with the development of CVD, such as obesity, hypertension, diabetes, and hyperlipidemia. Among them, hyperlipidemia plays an important role in inducing various types of CVD [1–3]. Hyperlipidemia plays an important role in the pathogenesis of CVDs such as hypercoagulability, hypofibrinolysis, and atherosclerosis [4].

To prevent poor quality of life and increased medical expenses because of CVD, efforts have been made to prevent the occurrence of CVD risk factors early on. Hyperlipidemia is a major risk factor for CVD. Serum low-density lipoprotein (LDL) cholesterol levels significantly affect the morbidity and mortality in CVD [5–7]. In addition, blood circulation disorder is a state in which the homeostasis of blood flow is disturbed, where vascular hemostasis and inhibitory action are balanced and normal blood flow is maintained [8, 9]. However, if the blood coagulation factor is excessively activated or platelet aggregation is promoted, blood flow homeostasis is disrupted and blood circulation is affected, resulting in thrombosis and embolism.

Thrombus, which is generated during the coagulation process, gets attached to the blood vessel wall and obstructs the flow of blood, increases the viscosity of blood, inhibits blood circulation, and interferes with the inflow of oxygen or nutrients into the tissue [10, 11]. Thrombosis and embolism due to hematogenous disorders cause serious CVDs such as myocardial infarction and stroke. Thus, the application of dietary therapy has been shown to improve hyperlipidemia and blood circulation disorders [12, 13].

This indirectly suggests that dietary changes will play an important role as one of the lifestyle adjustments. Many previous studies have focused on foods and nutrients that are expected to lower lipid levels and improve blood circulation and examined their potential in lowering lipid levels and alleviating blood circulation disorders after ingesting functional foods containing them. Eating habits and lifestyle, hyperlipidemia, hypertension, and diabetes are known as risk factors for blood circulation disorders [14–16].

Drugs used as anticoagulants include heparin injectables such as warfarin and apixaban and ingestible drugs such as edoxaban and rivaroxaban [17–19]. However, hemostatic agents traditionally used in medical practice have not always been effective in reducing the dangerous loss of blood because of hemorrhagic shock, dilute coagulation, or complications of thrombosis. It is especially important to develop new hemostatic agents with high efficacy and fewer side effects [20]. In anticoagulant research, the advantageous low toxicity of natural substances has attracted attention. Currently, far more studies are focusing on natural products and have discovered some natural products such as the ginkgo biloba leaf extract that can coagulate blood effectively [21–25].

Apart from its stronger antioxidation ability [26–28], *Vitis labrusca* shows other effects, such as hepatoprotection [29], cardioprotection, renal protection, and neuroprotection [30, 31]. The grape leaf extract (*V*. *labrusca* extract, VLE) was recently reported to improve the antiplatelet activity by inhibiting TXB(2) and serotonin when it was administered to SD rats for one week under normal conditions [32]. However, antiplatelet activity under normal conditions may imply an increased risk for both nonmajor and major bleeding. Therefore, it is essential to confirm the efficacy of antiplatelet activity in an experimental model in which blood coagulation is delayed, such as dyslipidemia and arteriosclerosis. The ability of HP01 to improve thrombotic and hyperlipidemia through the antioxidant activity on the hyperlipidemia model was investigated.

## 2. Materials and Methods

### 2.1. Chemicals

2, 2-Diphenyl-1-picrylhydrazyl (DPPH), vitamin C (50 *μ*M), Trolox, thiobarbituric acid, glutathione, glutathione reductase (GR), 5, 5-dithio-bis-(2-nitrobenzoic acid (DTNB), and sodium dodecyl sulfate (SDS) were purchased from Sigma (St. Louis, MO). CaCl_2_, prothrombin time (PT), and activated partial thromboplastin time (aPTT) were purchased from Siemens (USA). Ammonium dihydrogen phosphate (ADP) was purchased from Chrono-log (Pennsylvania, USA).

### 2.2. Extract of HP01

Sample extraction was conducted according to the method described by Kwon et al. [32] with minor modifications. The *Vitis labrusca* leaves were collected from Baekgu-myeon, Gimje-si, Jeollabuk-do, and Hwaseong-si, Gyeonggi-do, Republic of Korea, in April 2019. The botanical identification was authenticated by Dr. Hyoung-Kwon Cho of the Hanpoong Pharm. CO., Ltd., where the voucher specimen (#HP-01) was deposited. *V. labrusca* leaves (1 g) were extracted with 20 mL of acidified hydroalcoholic solvent (ethanol : water : acetic acid = 50 : 45 : 5 v : v : v) for 3 h at 80–90°C. After filtration, the residue was re-extracted as described above, two additional times. The combined filterates were the was centrifuged at 1,500 rpm at room temperature and then evaporated and lyophilized.

### 2.3. HPLC Estimation of Quercetin-3-*O*-glucuronide

Quercetin-3-*O*-glucuronide in HP01 was quantified by HPLC (Shimadzu LC-3030C3D, Kyoto, Japan) using a Waters Xbridge shield RP18 column, 4.6 mm I.D. × 150 mm, 3.5 *μ*m (Waters Co., Milford, MA, USA). In the HPLC quantitative analysis, the mobile phase was a mixture of 0.1% formic acid (A) and acetonitrile (B), with a gradient elution method: 0-40 min, a linear gradient from 15% B to 25% B; 40–60 min held at 15% B. Flow rate was 0.6 mL/min, injection volume was set to 10 *μ*L, and the peaks were detected at 330 nm. Equipment control, data acquisition, and integration were performed with Shimabzu software. The presence of quercetin-3-*O*-glucuronide was confirmed by retention time that was the same as that of the appropriate standards (EXTRASYNTHESE, France).

### 2.4. Antioxidant Activities of HP01

To confirm the antioxidant activity of HP01, DPPH radical-scavenging assay was performed using the method described by Klouwen [33]. The extracts were added to 0.2 mM DPPH and incubated for 30 min. Absorbance was measured at 520 nm. The inhibition rate was calculated as follows:

(1) $$\begin{array}{c} \text{Inhibition rate}=\left(1-\frac{A}{B}\right)\;\times \;100, \end{array}$$

where *A* is the absorbance of the sample, and *B* is the absorbance of the control.

Superoxide radical scavenging assay was also performed using the SOD assay kit—WST (Dojindo, Tokyo, Japan) according to the manufacturer's instructions. Optical density was measured at 450 nm. Vitamin C (50 *μ*M) or Trolox (500 *μ*g/mL) was used as the reference.

### 2.5. Animal Care

The animals used in this study were 5-week-old male Sprague–Dawley (SD) rats (initial weights 227.1 ± 37.9 g) that were purchased from Orient Bio Inc. (Kwangju, Republic of The platelet count was adjusted Korea). Rats were housed in specific pathogen-free (SPF) conditions at 21°C−24°C and between 40% and 60% relative humidity with a 12 h light-dark cycle. All animals were acclimatized for at least 1 week prior to the start of experiments. All studies were performed in accordance with the guidelines for animal experimentation by Wonkwang University and were approved (No. WK19-048). The rats were randomly selected and assigned to 5 groups (6 rats/group). One group served as normal (NOR) and was fed a NOR diet (5053 Pico Lab Rodent Diet, Purina LabDiet, CA, USA). The other 4 groups were fed a high-fat diet (HFD) (#D12492, New Brunswick, NJ, USA) containing 60% kcal from fat for 8 weeks without HP01. The HFD groups were additionally treated by gavage with HP01 (dissolved in distilled water) at 200 mg/kg and 400 mg/kg bodyweight for 4 weeks after hyperlipidemia induced. Gingko extract (GK, 50 mg/kg, p.o.) was used as the positive control. During the 12 weeks experimental period, mice had free access to food and water.

### 2.6. Preparation of Platelets

Blood was collected from the abdominal aortas using sodium citrate tubes (BD Biosciences, USA). The collected blood was centrifuged to obtain the platelet-rich plasma (PRP) and platelet-poor plasma (PPP). The platelet count was adjusted to 3 × 10^8^ platelets/mL for the platelet aggregation and anticoagulant assay.

### 2.7. Platelet Aggregation

Aggregation was measured turbidimetrically with slight modifications. Briefly, PRP was preincubated for 5 min at 37°C. Platelet aggregation was induced by adding ADP (25 mol/mL, Chrono-log, Havertown, Pennsylvania, USA). The amount of light transmitted through the sample was observed for 10 min at 37°C using an aggregometer (Model 700-2, Chrono-Log, Havertown, Pennsylvania, USA).

### 2.8. Anticoagulant Assay

aPTT (Pathrombin SL, Siemens Healthcare Diagnostics Inc., Marburg, Germany) and PT (Thromborel S, Siemens Healthcare Diagnostics Inc., Marburg, Germany) were determined according to the manufacturer's protocols. For the PT assay, 25 *μ*L of PRP was incubated for 1.5 min at 37°C. The assay reaction was started by adding 50 *μ*L of PT reagent and was observed for 3 min.

### 2.9. Thickness of the Carotid Artery

The carotid artery tissues were fixed in 10% formalin buffer solution and then routinely processed for paraffin embedding. The 4 *μ*m sections of each tissue were stained with hematoxylin and eosin and observed under an optical microscope (Zeiss Axioscope, Jena, Germany) at 200x magnification.

### 2.10. Determination of TG and TC

The levels of TG and total cholesterol in the blood and liver were determined using BioVision assay kits (Audit Diagnostics, Cork, Ireland) and Asan cholesterol enzymatic kit (Asan, Seoul, Korea) according to the manufacturer's instructions. The lipids were extracted from the liver samples following the modified method described by Folch et al. Briefly, total lipids were extracted from the liver samples in the homogenizing buffer (0.154 M KCl, 50 mM Tris-HCl, and 1 mM EDTA).

### 2.11. Determination of Hepatic Damage and Antioxidant Activity

Serum isolated from the collected blood samples was used for determining the AST and ALT levels according to the method described by Reitman and Frankel [34]. MDA, the end product of lipid peroxidation, was measured using a slightly modified thiobarbituric acid reactive substance assay [35]. The total GSH was measured according to the method described by Griffith et al. [36]. Antioxidant enzyme activity was analyzed using specific enzyme assay kits (Cayman Chemical, Michigan, USA) in hepatic cytosol. Superoxide dismutase (SOD) activity (Cu/Zn, Mn, and FeSOD) was measured by diluting samples to 1 : 1,000. Catalase (CAT) activity was measured by 4-amino-3-hydrazino-5-mercapto-1, 2, 4-triazole (purpald) in a sample diluted to 1 : 1,000. Glutathione peroxidase (GPx) assay was performed in the presence of glutathione (GSH) and oxidized glutathione (GSSG).

### 2.12. Statistical Analysis

All data are expressed as mean ± SD of at least three independent experiments. Significant differences were compared using repeated measures ANOVA followed by the Newman–Keuls multiple range test. Statistical significance was defined as *P* < 0.05. All statistical analyses were performed using GraphPad Software, Inc. (San Diego, CA).

## 3. Results

### 3.1. Proximate Composition of HP01

Proximate analysis involves the determination of major components of an extract, including carbohydrate, crude fat, crude protein, moisture, ash, and sodium. The proximate composition of HP01 accessions is presented in Table 1.

### 3.2. Chromatographic Analysis of the Quercetin-3-*O*-glucuronide Profile in HP01

**Figure 1:**
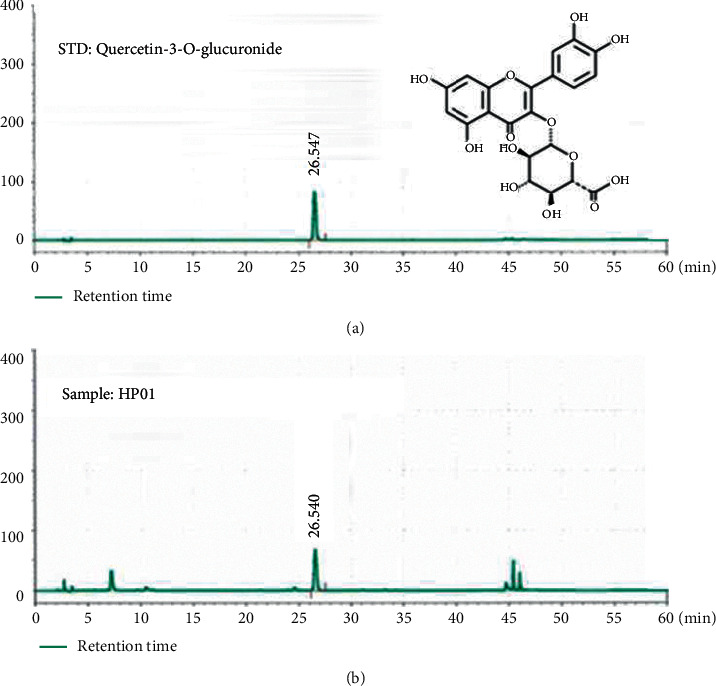
High-performance liquid chromatography analysis of quercein-3-*O*-glucironide in HP01. The marker compound of HP01 was analyzed using high-performance liquid chromatography.

Quercetin-3-*O*-glucuronide content in HP01, as determined by HPLC, was 14.13 ± 2.83 mg/g. Peak identification was based on the UV spectrum and the retention times of the quercein-3-*O*-glucuronide standard used in our laboratory. HPLC analysis at 330 nm revealed peaks at 26.54 minutes and 26.56 minutes for the standard and HP01, respectively (Figure 1).

### 3.3. Effects of HP01 on Radical Scavenging Activity

**Figure 2:**
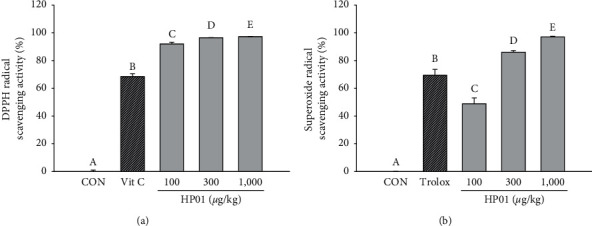
Effects of HP01 on antioxidant activities. (a) Hydroxyl and (b) superoxide radical scavenging activities of HP01. Data are shown as mean ± SD of changes in the DPPH and superoxide radical scavenging activity. The results are presented as the mean ± SD. Values with different letters (*A, B, C, D,* and *E*) are significantly different one from another (one-way ANOVA followed by the Newman–Keuls multiple range test, *P* < 0.05).

DPPH radical scavenging activity was determined to confirm the antioxidant effects of HP01. HP01 increased DPPH radical scavenging activity in a concentration-dependent manner and exhibited up to 94.3% scavenging activity at 100 *μ*g/mL and 500 *μ*g/ml. Ascorbic acid (50 *μ*M) was used as the positive control. SOD-like activity measurements revealed that the positive control Trolox (500 *μ*g/mL) showed a scavenging activity of 56.2%. HP01 showed a significant difference in scavenging activity compared with the control, and a radical scavenging activity of 51.9% was confirmed at 500 *μ*g/mL. These data suggest a dose-dependent antioxidant effect of HP01 (Figure 2).

### 3.4. Effects of HP01 on Bodyweight, Food Intake, and Tissue Weights

The average bodyweight of the rats fed a HFD for 8 weeks was 665.0 ± 21.8 (g), and the groups were repositioned, and the candidates were administered for 3 weeks.

No significant differences in bodyweight were observed among groups during the experimental period. The weight gain in rats of the GK (62.1 ± 9.8 g) and 200 mg/kg HP01 (73.9 ± 9.8 g) groups, compared to that of the HFD group (93.8 ± 10.7 g), showed a tendency to decrease by 33.8% and 21.2%, respectively.

The HFD group consumed 101.3 ± 16.3 kcal (21.2 ± 3.1 g) of diet (HFD, 5.1 kcal = 1 g) on an average. No change in food intake because of HP01 was observed.

After 4 weeks of administration, the experimental animals were anesthetized, and then, the pDEXA equipment was used to measure the bone density of the thigh, body fat mass of the whole body, and muscle mass of the calf muscle part. There was no increase in bone density and calf muscle weight because of HP01 administration. However, HP01 (400 mg/kg) significantly attenuated the HFD-induced increase in fat mass (*P* < 0.05) (Table 2). There was no change in the weight of the liver, spleen, kidney, testis, heart, and lung because of HP01 administration. Epididymal fat weight showed a 37.2% decrease in the GK group, and HP01 caused a decrease of 33.1% and 23.9% at 200 mg/kg and 400 mg/kg, respectively, compared to that of the control group (Table 3).

### 3.5. Effects of HP01 on Thickness of the Carotid Artery

**Figure 3:**
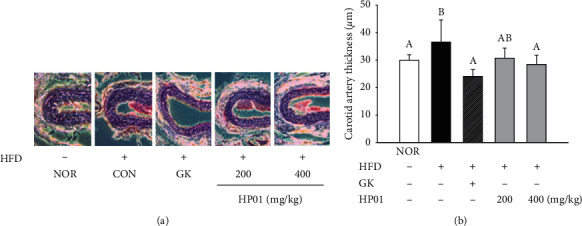
Effects of HP01 on carotid artery thickness in hyperlipidemic rats. HP01 was administered orally once a day for 4 weeks in hyperlipidemic rats. Gingko extract (GK, 50 mg/kg, p.o.) was used as the positive control. (a) Hematoxylin and eosin-stained sections of carotid artery tissues (original magnification ×200). (b) Measurement of carotid artery thickness. Values are reported as the mean ± standard deviation. Values with different letters (*A* and *B*) are significantly different one from another (one-way ANOVA followed by the Newman–Keuls multiple range test, *P* < 0.05).

The carotid blood vessels of the SD rats were isolated to observe changes in wall thickness of the blood vessels following HFD intake. The GK (24.0 ± 2.5 *μ*m) and HP01 400 mg/kg (28.4 ± 3.4 mm) groups showed significant decreases in wall thickness of 34.2% and 22.3%, respectively, compared to that of the HFD group (36.5 ± 8.0 *μ*m) (Figure 3).

**Figure 4:**
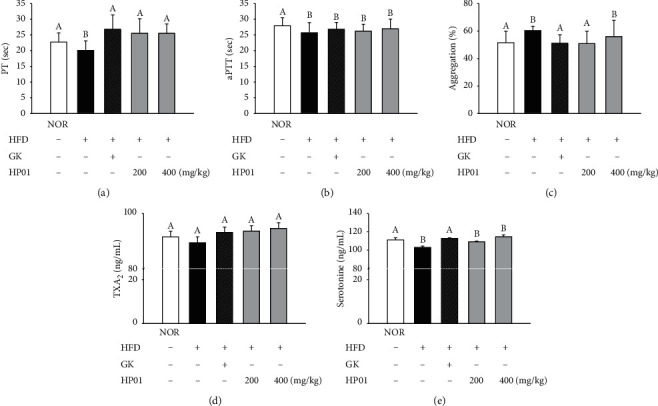
Effects of HP01 on platelet activity in hyperlipidemic rats. HP01 was administered orally once a day for 4 weeks in hyperlipidemic rats. Gingko extract (GK, 50 mg/kg, p.o.) was used as the positive control. The platelet activity shown as (a) PT, (b) aPTT, and (c) platelet aggregation. Serum levels of (d) TXA2 and (e) serotonin. Values are reported as mean ± standard deviation. Values with different letters (*A* and *B*) are significantly different one from another (one-way ANOVA followed by the Newman–Keuls multiple range test, *P* < 0.05).

PT was shortened by HFD intake, and HP01 treatment at 200 mg/kg and 400 mg/kg improved PT by 5.5 and 5.4 seconds, respectively. aPTT was significantly delayed by 1.09 s by GK and did not show an increase with HP01 treatment. The effect of blood coagulation on HP01 was confirmed by the impedance method, and platelet aggregation was confirmed by the optical method. The test was performed on PRP, and ADP was used as an agonist. Platelet aggregation, which was increased by HFD intake, decreased by 25.9% in the GK group, and a significant decrease was observed in the HP01 200 mg/kg group. Serum TXA2 and serotonin levels were measured, but no significant increase or decrease was observed in the HFD fed groups compared to those in the control group (Figure 4).

### 3.6. Effects of HP01 on Lipid Profile

To examine the effects of HP01 on the lipid profile, we determined the levels of TG and TC in the liver and serum. Administration of HFD was found to result in significantly increased hepatic TG and TC levels in the HFD group. The HP01 group treated with SBS showed markedly lower levels of TG and TC in the liver. In addition, after HFD intake for 12 weeks, hepatic total cholesterol levels increased compared to those of the NOR group. However, hepatic total cholesterol levels in the HP01 group decreased by approximately 36.2% compared to the levels in rats on a HFD. However, no significant change in blood TG levels was observed. These results can be attributed to the ability of HP01 to effectively suppress the accumulation of hepatic TG and TC in HFD-fed rats (Table 3).

### 3.7. Effects of HP01 on Hepatic Antioxidant Activity and Damage

**Figure 5:**
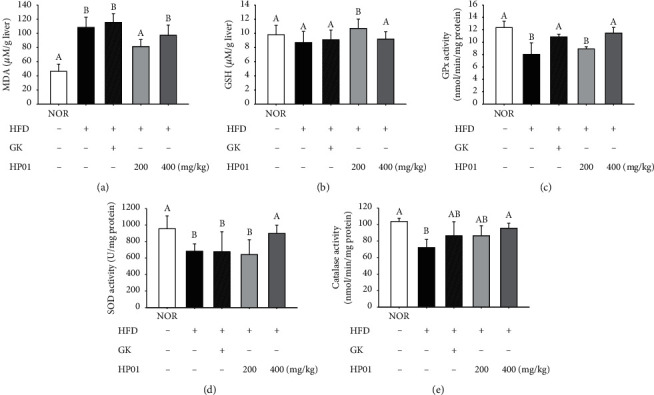
Effects of HP01 on the hepatic protection in hyperlipidemic rats. HP01 was administered orally once a day for 4 weeks in hyperlipidemic rats. Gingko extract (GK, 50 mg/kg, p.o.) was used as the positive control. The hepatic levels of (a) MDA, (b) GSH, (c) GPx, (d) SOD, and (e) catalase. Values are reported as the mean ± standard deviation. Values with different letters (*A, B,* and *C*) are significantly different one from another (one-way ANOVA followed by the Newman–Keuls multiple range test, *P* < 0.05).

**Figure 6:**
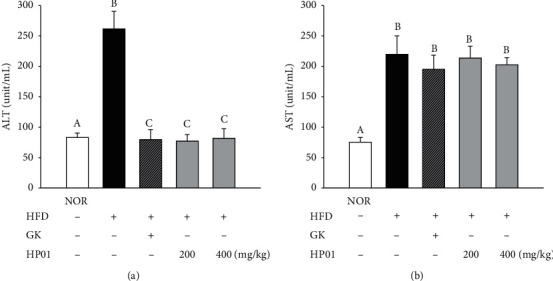
Effects of HP01 on the hepatic protection in hyperlipidemic rats. HP01 was administered orally once a day for 4 weeks in hyperlipidemic rats. Gingko extract (GK, 50 mg/kg, p.o.) was used as the positive control. The serum levels of (a) ALT and (b) AST. Values are reported as the mean ± standard deviation. Values with different letters (*A, B,* and *C*) are significantly different one from another (one-way ANOVA followed by the Newman–Keuls multiple range test, *P* < 0.05).

MDA, an indicator of oxidative stress, increased with HFD intake. However, 200 mg/kg and 400 mg/kg of HP01 decreased oxidative stress by 23.7% and 7.4%, respectively, and a significant difference was observed with HP01 200 mg/kg (Figure 5(a)). When HP01 was administered at 200 mg/kg and 400 mg/kg, GSH levels decreased because of a significant increase in oxidative stress by 21.6 and 10.9%, respectively. Similarly, the HFD group exhibited significantly declined activity of hepatic GPx (Figure 5(c)), SOD (Figure 5(d)), and CAT (Figure 5(e)) when compared with the control group. 400 mg/kg of HP01 administration significantly improved the activity of GPx, SOD, and CAT by 42.9%, 31.6%, and 32.4%, respectively, in the liver of the HFD group. Increased ALT levels due to HFD intake decreased by 69.3% in the GK group and significantly decreased by 70.2% and 68.4% in the 200 mg/kg and 400 mg/kg HP01 groups, respectively. However, no decrease in AST was observed with HP01 treatment (Figure 6).

## 4. Discussion

Grapes are rich in organic acids such as tartaric acid, malic acid, citric acid, and vitamins *A*, *B*, and *C*. In particular, it has been reported that grapes contain various polyphenolic components including flavonoids, such as anthocyanins, catechin, and resveratrol [37, 38]. It is known that polyphenolic components in grapes are mainly present in the grape skin and seeds and are distributed not only in the edible parts but also in the leaves. These polyphenolic components are known to be effective in preventing CVD, cancer, arteriosclerosis, aging, and thrombosis and have been reported to be involved in exerting antioxidant effects through antioxidation of serum LDL. When problems such as dietary habits and lifestyles act in combination to increase oxidative stress in the body, the risk of developing chronic carcinoma, vascular disease, and metabolic syndrome increases. These phytochemicals protect the body from oxidative damage inflicted by reactive oxygen species (ROS) through their antioxidant effects and free radical scavenging. These phytochemicals possess antioxidant effects and free radical scavenging that protects the body against oxidative damage produced by ROS [39–42]. In the present study, HP01 showed a high antioxidant effect.

Polyphenols/flavonoids, which contain two or more aromatic rings, are contained in various fruits and legumes; according to the studies by Ahmad et al. [43], 5,000 flavonoids have been found so far. Flavonoids provide color and flavor to fruits and vegetables [43, 44].

In case of natural products, the components of the extract may vary depending on the harvest time and place; thus, a marker compound was selected to ensure the quality of the HP01 extract. According to Singh and Zhao [45], markers are chemically defined constituents of a herbal drug that are of interest for the quality control purposes, independent of whether they have any therapeutic activity or not. We have selected quercetin-3-*O*-glucuronide as the marker compound of HP01.

In this study, we demonstrated for the first time that HP01 can improve blood circulation by suppressing coagulation and platelet activation and reducing lipid levels in SD rats fed a HFD for 8 weeks. Hyperlipidemia refers to the concentration of one or several components exceeding normal levels in the hepatic and blood lipid profiles, such as cholesterol, triglyceride, phospholipid, and free fatty acid [46]. As these variations are frequently observed in diseases such as obesity, insulin resistance, and diabetes, they can be used as biomarkers of the diseases.

Ageno et al. reported that mean triglyceride levels were higher and HDL-C levels were lower in venous thrombosis patients than in control patients [47]. Morelli et al. have previously shown that lipid-lowering drugs (statins, most notably rosuvastatin) are associated with a decreased risk of venous thrombosis, which might indicate a possible role of lipids in the pathophysiology of venous thrombosis [48]. Zajtchuk and Zajtchuk had reported the data revealing that patients with high triglyceride levels also had a high incidence of low antithrombin activity and increased platelet aggregation [49]. It is likely that hyperlipidemic patients are more prone to thrombosis of diseased coronary arteries.

Therefore, we selected the increase in TG and total cholesterol as the biomarkers for thrombosis and conducted a study on the blood circulation improving function of HP01.

We administered a 60% HFD to induce hyperlipidemia before HP01 administration and measured TG levels at 2 week intervals. No change was observed in blood TG and total cholesterol levels of SD rats fed a HFD by week 8. However, hepatic TG and total cholesterol levels showed a significant increase of 3.37-fold and 5.7-fold, respectively, compared to that of the normal groups. These results were similar to the results obtained from previous study by Chiu et al. where the hepatic TG and total cholesterol were measured after 8 weeks of ingesting HFD [50]. HP01 was administered from 8 weeks, and the TG and total cholesterol concentration showed a significant difference compared to that of the normal group.

In the present study, high-fat feeding induced a significant increase in fat weight, which represents adipocyte hypertrophy. Our results confirm the suppressive effect of -HP01 on HFD-induced adiposity. No anatomical abnormalities were found between the NOR group and the HFD group. These results showed that the color and pattern of each organ other than the liver were not different from those of the normal group, indicating that hyperlipidemia and other diseases were not induced.

In our experiments, there was no change in the amount of lipids in serum, but an increase in the amount of the liver tissue lipids was observed. In animals that induced hyperlipidemia by HFD during a period of less than 12 weeks, blood did not show any changes in the blood lipid profile but only differences in lipids in the liver tissue [16, 51–54]. According to the previous studies by Kim et al., HFD must be taken for at least 15 weeks for changes in blood lipids to occur [55]. The changes in lipid in the hepatic tissue following the induction of hyperlipidemia were decreased by the administration of HP01 [56].

Increased vascular endocardial thickness is an important biomarker for diagnosing CVD. The arterial plaque with hypertrophy was significantly increased by lipid accumulation in HFD-induced hyperlipidemia. Our results also have demonstrated HFD-induced hypertrophic changes in the aortic wall. A significant improvement effect was confirmed in the HP01 group.

In hyperlipidemia, altered lipid levels disrupt the balance of the procoagulant and anticoagulant pathways. In addition, it induces the activation of procoagulation enzymatic complexes (factor Xa and factor Xa/Va-mediated factor VII). Our results show significantly shorter PT and aPTT in hyperlipidemic rats than in normal rats.

Blood clotting occurs through a series of proteolytic reactions, including the stepwise activation of coagulation factors I–XII. Some of these factors are activated by two pathways: the extrinsic pathway and the intrinsic pathway. In general, aPTT is used to determine the overall efficiency of the extrinsic clotting pathway, and PT is an intrinsic clotting pathway screening test. Our results showed that HP01 prolonged PT in hyperlipidemic rats. However, it did not affect aPTT. High blood lipid levels are associated with increased clotting factors [57]. Kim et al. have reported a significant correlation between total cholesterol and triglyceride levels and procoagulant factors II, VII, IX, and X, except fibrinogen, implying that the elevation of procoagulant factors may contribute to shorter PT values in subjects with high blood lipids [58]. Because there was no association of factor VIII with blood lipid levels in our study, subjects with HP01 also did not show a delayed aPTT value compared to those with hyperlipidemia. These findings suggest that HP01 contributes to improving the blood flow by regulating the extrinsic clotting pathway.

Serotonin and TXA2 are known as the most typical vascular regulators that mediate vasoconstriction because of platelet aggregation. TXA2 is a metabolite of arachidonic acid and is known to regulate platelet aggregation and vasomotor responses along with nitric oxide. In the study by Kim et al., study, the GBx administration reduced the HFD-induced TXA2 and serotonin levels [59]. However, the results of our experiments did not show a significant decrease in increased TXA2 and serotonin by HP01. These results imply that HP01 is not associated with vasomotor responses.

Hyperlipidemia induces oxidative stress because of lipid accumulation. Monteiro et al. reported that metabolic abnormalities as a consequence of HFD in rats cause platelet hyperaggregability involving enhanced intraplatelet ROS production and decreased NO bioavailability that are accompanied by potential defects in the prosthetic heme group of sGC [60]. Our data showed that ADP-induced platelet aggregation was significantly higher in the HFD group. HP01 significantly downregulated the aggregation compared to that of the HFD group. These results can be attributed to the antioxidant effect of HP01.

Matsuzawa-Nagata et al. showed that pathways involved in the mitochondrial respiratory chain were downregulated in both the liver and adipose tissue of mice fed HFD [61]. High mitochondrial *β*-oxidation rates may help metabolize excess free fatty acid (FFA), but a large number of electrons entering the respiratory chain can cause abnormal oxygen depletion. The production of mitochondrial ROS through which electrons flow can be increased. This suggests that the respiratory dysfunction can increase the production of mitochondrial ROS. Hyperlipidemia results in unbalance between oxidation and antioxidation and produces a large number of oxygen free radicals in vivo. Furthermore, oxygen-free radicals translate to MDA. Oxygen-free radicals and MDA result in the injury of vascular endothelial cell and promote the formation and development of atherosclerosis. The activities of GSH-Px, SOD, and CAT directly reflect the ability to scavenging oxygen-free radicals. Santos et al. reported that *V*. *vinifera* L. grape skin extract (ACH09) prevented the increase in MDA and protein carbonylation levels and reduced the antioxidant activity of SOD, CAT, and GPx, suggesting an important protective effect against oxidative stress that may contribute toward the improvement of mitochondrial function and reduction of lipid accumulation in the liver [53]. Also, our research showed that HP01 administration significantly decreased hepatic MDA levels and increased the total GSH, SOD, CAT, and GPx activity compared to those of the HFD group. These results indicate the strong antioxidant capacity of HP01. Aminotransferase activity is altered during the course of lipid metabolism because of fat accumulation. Measurement of the liver damage because of interstitial fat accumulation is important for the diagnosis of NAFLD. Therefore, AST and ALT levels were measured as indicators of liver damage. In the current study, AST and ALT levels showed a more pronounced increase in rats fed HFD than in rats fed ND. However, HP01 supplementation significantly reduced ALT and AST levels, indicating a strong hepatoprotective effect in HFD-induced liver injury. Taken together, all of the above results show that HP01 exhibits remarkable free radical scavenging activity both in vitro and in vivo, as well as hypolipidemic and hepatoprotective activities in vivo. Despite these findings, the precise mechanism by which the HP01 exhibited its antioxidant and hypolipidemic effects cannot be concluded from these data which is a limitation of this study. Hence, further studies using the HP01, as well as constituents, on molecular pathways such as lipid synthesis and antioxidant regulator are highly required.

## 5. Conclusions

Administration of HP01 improved blood circulation and ameliorated lipid-related disorders in the hyperlipidemia model. These effects are considered that potent antioxidant activity of HP01 may be directly or indirectly responsible for its hypolipidemic properties. The HP01 might be considered as an alternative functional food or pharmaceutical in the prevention and treatment of hyperlipidemia. As a powerful antioxidant, HP01 needs further research as a therapeutic agent for the treatment of hyperlipidemia.

## Figures and Tables

**Table 1:** Proximate composition of HP01.

Composition (unit)	Amount
Gross energy (kcal/100 g)	343.10 ± 5.1
Carbohydrate (%)	72.87 ± 2.4
Crude fat (%)	2.74 ± 0.7
Crude protein (%)	6.74 ± 1.8
Moisture (%)	8.91 ± 1.7
Ash (%)	8.74 ± 0.8
Sodium (mg/100 g)	79.96 ± 5.1

**Table 2:** Effects of HP01 on bodyweight, food consumption, fat mass, and relative epididymal fat in hyperlipidemic rats.

Groups/factors			Bodyweight						Food intake (kcal)		FAT			
			Initial		Final		Gain				Mass (%)		Relative epididymal fat (%)	
NOR			545.2 ± 63.8	a	617.6 ± 61.4	a	78.0 ± 20.1	a	87.5 ± 0.9	a	45.1 ± 12.1	a	2.1 ± 0.5	a
HFD	CON		668.5 ± 27.3	b	762.3 ± 25.5	b	93.8 ± 10.7	a	101.3 ± 16.3	a	62.8 ± 4.0	b	4.6 ± 1.2	b
	GK		664.7 ± 23.6	b	705.8 ± 23.9	b	62.1 ± 15.2	b	88.1 ± 18.5	a	49.4 ± 6.7	a	2.9 ± 0.3	a
	HP01 (mg/kg)	200	662.7 ± 23.6	b	736.0 ± 23.4	b	73.9 ± 15.2	a	91.4 ± 18.5	a	50.8 ± 6.8	b	3.1 ± 0.3	a
		400	663.7 ± 21.2	b	773.0 ± 17.3	b	86.8 ± 7.2	a	92.7 ± 10.1	a	47.6 ± 2.5	b	3.5 ± 0.5	b

HP01 was administered orally once a day for 4 weeks in hyperlipidemic rats. Gingko extract (GK, 50 mg/kg, p.o.) was used as the positive control. The results are presented as the mean ± SD. Values with different letters (a and b) are significantly different one from another (one-way ANOVA followed by the Newman–Keuls multiple range test, *P* < 0.05).

**Table 3:** Effects of HP01 on lipid profile in hyperlipidemic rats.

Group			Hepatic (mg/g liver)				Serum (mg/dL)			
			TG		Total cholesterol		TG		Total cholesterol	
NOR			2.8 ± 1.1	a	2.2 ± 0.3	a	351.8 ± 168.2	a	94.7 ± 29.3	a
HFD	CON		16.0 ± 2.9	b	13.4 ± 7.4	b	368.9 ± 166.2	a	127.5 ± 36.9	a
	GK		12.2 ± 3.7	a	7.6 ± 3.1	a	512.5 ± 114.2	a	102.6 ± 20.9	a
	HP01 (mg/kg)	200	14.7 ± 2.9	a	8.6 ± 1.0	a	593.3 ± 266.8	a	138.7 ± 31.2	a
		400	10.8 ± 1.6	c	12.5 ± 6.8	a	587.2 ± 148.0	a	130.7 ± 30.0	a

HP01 was administered orally once a day for 4 weeks in hyperlipidemic rats. Gingko extract (GK, 50 mg/kg, p.o.) was used as the positive control. Values are reported as mean ± standard deviation. Values with different letters (a, b, and c) are significantly different one from another (one-way ANOVA followed by the Newman–Keuls multiple range test, *P* < 0.05).

## Data Availability

The datasets used and/or analyzed during the current study are available from the corresponding author upon request.

## Abbreviations

HFD: High-fat diet
PT: Prothrombin time
aPTT: Activated partial thromboplastin time
TXB: Thromboxane *B*
AST: Aspartate aminotransferase
ALT: Alanine aminotransferase
TG: Triglyceride
MDA: Malondialdehyde
GSH: Glutathione
CVD: Cardiovascular disease
LDL: Low-density lipoprotein
PGI2: Prostaglandin I2
DMEM: Dulbecco's modified eagle medium
SD: Sprague–Dawley
SPF: Specific pathogen-free
NOR: Normal
HPLC: High-performance liquid chromatography
DPPH: 2,2-Diphenyl-1-picrylhydrazyl
SOD: Superoxide dismutase
PRP: Platelet-rich plasma
PPP: Platelet-poor plasma
ADP: Ammonium dihydrogen phosphate
HCl: Hydrogen chloride
UV: Ultraviolet
GK: Ginkgo extract
DEXA: Dexamethasone
ROS: Reactive oxygen species
VLE: *Vitis labrusca* extract
GBx: Ginsenoside berry
NO: Nitric oxide
FFA: Free fatty acid.
